# Exploring the Impact of Niacin Intake on Cardiovascular Outcomes: A Comprehensive Analysis Using NHANES Data (2003–2018)

**DOI:** 10.31083/j.rcm2511410

**Published:** 2024-11-20

**Authors:** Lishi Shao, Aihua Zhi, Manning Li, Yang Zhang, Shaohui Jiang, Jun Zhang, Ke Yang, Enze Yang, Xiankang Zhu, Yuanou Cheng, Yi Sun

**Affiliations:** ^1^Department of Radiology, Fuwai Yunnan Cardiovascular Hospital, 650102 Kunming, Yunnan, China; ^2^Department of Cardiac Surgery, Fuwai Yunnan Cardiovascular Hospital, 650102 Kunming, Yunnan, China; ^3^Department of Vascular Surgery, Kunming Children’s Hospital, 650034 Kunming, Yunnan, China; ^4^Department of Ultrasound, Fuwai Yunnan Cardiovascular Hospital, 650102 Kunming, Yunnan, China; ^5^Department of Anesthesiology, Fuwai Yunnan Cardiovascular Hospital, 650102 Kunming, Yunnan, China

**Keywords:** niacin, CVD, all-cause, prevalence, mortality, female

## Abstract

**Background:**

The relationship between cardiovascular outcomes and niacin consumption levels remains unclear. This study aimed to examine the correlation between niacin intake and the incidence of cardiovascular disease, as well as the mortality rates associated with cardiovascular disease and other causes.

**Methods:**

From 2003 to 2018, we continually investigated updated information from the National Health and Nutrition Examination Survey. Based on the quartiles of niacin intake levels, four distinct categories of participants were established: Q1 (<14.646 mg), Q2 (14.646–21.302 mg), Q3 (21.302–30.401 mg), and Q4 (>30.401 mg). Baseline variable differences were assessed employing the Chi-Square and Student's *t*-tests. A weighted logistic regression with multiple variables was used to determine the association between niacin intake and cardiovascular disease prevalence. Hazard ratios (HRs) and 95% confidence intervals (CIs) for all-cause death and cardiovascular disease were determined utilising hazard regression models. Kaplan–Meier curves were used to compare survival probability between the high and low niacin intake groups, and dose-response linear relationships were evaluated with restricted cubic splines.

**Results:**

The cohort analysis included 80,312 participants for the assessment of niacin intake. Comparing the Q1 dataset to the Q4 dataset in the overall population, weighted Cox regression analysis showed a negative association with all-cause mortality (95% CI: 0.71–0.96, HR: 0.82) and mortality owing to cardiovascular disease (95% CI: 0.67–0.96, odds ratio (OR): 0.80). Sex-based subgroup analysis revealed a detrimental correlation between niacin use and overall mortality in females (Q4 cohort: 95% CI: 0.62–0.97, HR: 0.78) but not in males. Additionally, the Q3 (95% CI: 0.59–0.94, HR: 0.75) and Q4 (95% CI: 0.51–0.97, HR: 0.7) groups exhibited a negative association with female cardiovascular disease mortality compared to the Q1 group. Niacin intake was not significantly correlated with prevalence, all-cause mortality, or death from cardiovascular disease in males.

**Conclusions:**

Higher niacin consumption was correlated with a decreased risk of cardiovascular disease and death from all causes across the entire study population. Nevertheless, only females, and not males, exhibited a beneficial effect on mortality.

## 1. Introduction

Niacin, or vitamin B3, is an essential nutrient obtained from dietary sources, such as meat, fish, cereals, and vegetables [1]. In gram quantities, niacin has been shown to positively affect lipid profiles, lowering triglycerides and low-density lipoprotein cholesterol (LDL-C) levels while raising high-density lipoprotein cholesterol (HDL-C) levels [2, 3, 4]. However, the change in HDL-C caused by niacin is logarithmic, whereas the change in LDL-C is linear [5]. Niacin lowers serum LDL-C through multiple mechanisms, such as inhibiting the peripheral mobilisation of free fatty acids [6], thereby reducing the substrates for the hepatic synthesis of triglycerides and very low-density lipoprotein (VLDL) particles [7], which, in turn, reduces the hepatic conversion of VLDL particles to LDL particles. Additionally, niacin directly interferes with the enzymatic processes that mediate the conversion of VLDL-C to LDL-C [7] and reduces triglyceride synthesis and hepatic lipoprotein secretion by inhibiting diacylglycerol acyltransferase 2 [8]. However, skin flushing is a typical adverse effect associated with niacin. The recommended daily dose for individuals is typically between 15 and 20 mg [9], with pharmacological dosages of up to 3000 mg/day being well-tolerated in treating dyslipidaemia [10].

Historically, the 1975 Coronary Drug Project first recognized niacin’s potential to reduce atherosclerotic cardiovascular events [11]. Next, several early investigations suggested that gram-level niacin therapy could impact cardiovascular risk in secondary prevention, particularly for individuals already affected by cardiovascular disease (CVD) [12]. However, the cardiovascular benefits of niacin therapy remain a subject of debate. The Framingham Heart Study also provided epidemiological support for niacin’s potential to influence CVD through lipid modification pathways, suggesting an inverse relationship between CVD incidence and HDL-C levels [13, 14]. However, the Heart Protection Investigation 2-Treatment of HDL for Reducing the Rate of Vascular Breaks (HPS2-Thrive) investigation, which involved 35,301 patients primarily in secondary prevention trials, was meta-analysed in 2014 and found that adding niacin to statin medication did not significantly alter the death rates from stroke, coronary heart disease, nonfatal myocardial infarct, or all causes [15, 16]. The inconsistent protective effects of niacin observed in existing studies may be attributed to the trial design. Furthermore, the extent to which niacin contributes to these benefits remains unclear. The Institute for Clinical Systems Improvement does not recommend the co-treatment of niacin and statins owing to an elevated risk of side effects without a corresponding decrease in cardiovascular outcomes [17]. However, in the United States, many patients continue to use niacin for other indications approved by the Food and Drug Administration [18].

However, data on the association between niacin levels and CVD mortality are limited. To address these research gaps, this study explored the potential relationship between niacin intake and the risk of all-cause and CVD mortality. This study aimed to provide dietary recommendations that may contribute to improved CVD management.

## 2. Methods

### 2.1 Study Population

**Fig. 1.**
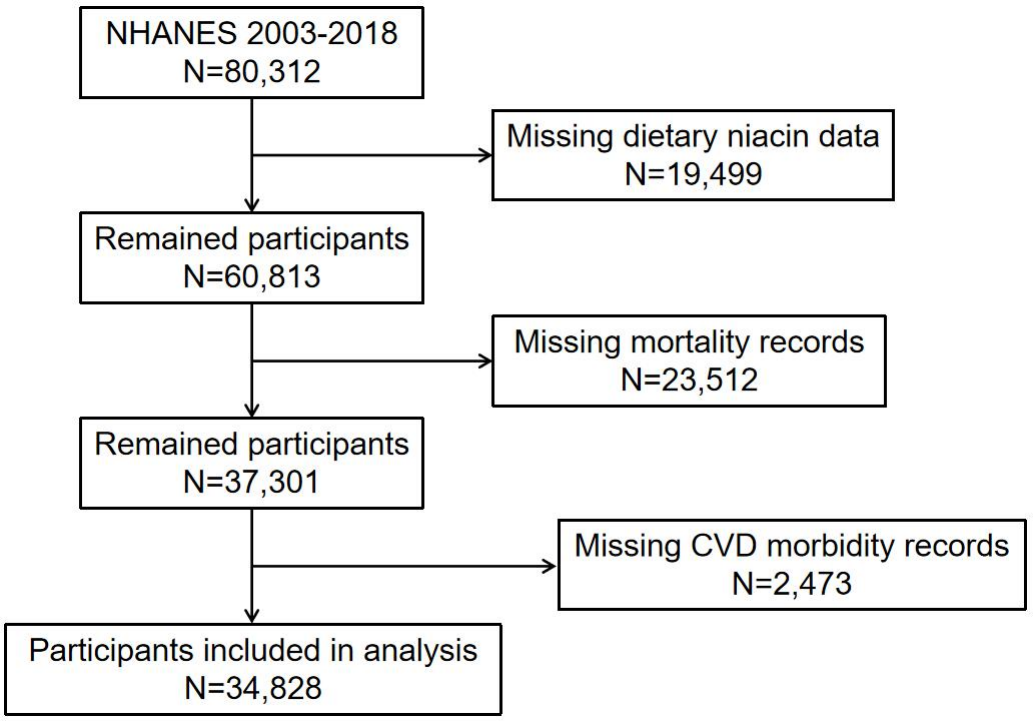
**The flow chart of participant selection**. NHANES, National Health and Nutrition Examination Survey; CVD, cardiovascular disease.

Participant information from the National Health and Nutrition Examination Survey (NHANES), a cross-sectional survey representing all non-institutionalised civilian populations in the United States, was used in this study. The National Centre for Health Statistics (NCHS), a branch of the Centre for Disease Control and Prevention branch, oversees the NHANES Life and Health Statistics Collection Project. A sophisticated multistage probability sampling strategy was used to ensure that the outcomes could be applied to other populations with an oversampling of older adults and members of underrepresented groups [19]. Fig. 1 illustrates the participant selection process followed in this study. NHANES data was collected from cycles 2003–2004 (n = 10,122), 2005–2006 (n = 10,348), 2007–2008 (n = 10,149), 2009–2010 (n = 10,537), 2011–2012 (n = 9756), 2013–2014 (n = 10,175), 2015–2016 (n = 9971), and 2017–2018 (n = 9254). After excluding records with missing niacin intake information, our primary analysis included 34,828 participants (**Supplementary Table 1**). The Ethics Review Board of NCHS granted ethical approval for including human subjects in NHANES. The subjects were informed about the study, and their consent was obtained.

### 2.2 Exposure and Outcomes

The What We Eat in America component of the NHANES study used 24-h food recall interviews to gauge individuals’ nutritional intake. A second phone interview was conducted 3–10 days after the first interview, which took place at the mobile examination centre. All participants were assessed for their ability to complete the dietary interviews. The Department of Agriculture clarified the intake of micronutrients, antioxidants, and total energy [20], while a questionnaire interview assessed dietary supplement intake in the past month, including frequency, dosage, and consumption duration. CVD was verified by self-reported clinician diagnoses obtained through individual interviews using an established medical condition questionnaire. This study focused on two significant outcomes: all-cause mortality, and CVD-related prevalence and mortality. NHANES data were probabilistically matched to National Death Index certificate records to evaluate mortality status, enabling the determination of participants’ vital status and analysis of mortality outcomes [21].

### 2.3 Covariates

The NHANES collected data on several demographic and sociodemographic factors using standardised questionnaires. These included age at participation, sex, race (categorised as Non-Hispanic Black, Mexican American, Non-Hispanic White, Other Hispanic, and Other Race), education level (defined as below the 9th grade, grades 9th–11th, includes the 12th grade without a diploma, graduates of high school, general educational development (GED) holders, or equivalent, associate degree, and bachelor’s degree or higher), and marital status (divorced, never married, widowed, married, separated, or surviving with a spouse). Body mass index (BMI), computed by dividing weight (kg) by height squared (m^2^), is a health measure. Hypertension was considered to be present if at least one of the following conditions was met: diastolic blood pressure (DBP) ≥80 mmHg, systolic blood pressure (SBP) ≥130 mmHg, history of hypertension, or current use of antihypertensive medication [22]. A self-reported diagnosis of diabetes was defined as haemoglobin A1c ≤6.5% or fasting blood glucose ≤7.0 mmol/L [23]. Stroke, congestive heart failure, heart attack, coronary heart disease (CHD), and angina pectoris are among the CVD-related death criteria [24]. Lifestyle factors were also assessed. Alcohol consumption was ascertained by enquiring as follows: ‘Over the previous 12 months, what was the average number of drinks consumed on days when alcohol-based drinks were consumed?’ Smoking habits were classified as never smoker, occasional smoker, or current smoker. Multiple imputation techniques were employed to address missing values, ensuring a complete dataset for analysis.

### 2.4 Statistical Analysis

Statistical analyses followed NHANES guidelines [25], accounting for the complex sampling design and sample size. Baseline and nutritional characteristics were examined across quartiles of niacin intake level: Q1 (<14.646 mg), Q2 (14.646–21.302 mg), Q3 (21.302–30.401 mg), and Q4 (>30.401 mg). The participants were grouped into cohorts based on these quartiles. Survey-weighted linear regression was employed for continuous variables to calculate *p*-values and means (95% confidence interval [CI]). Categorical variables were analysed via survey-weighted chi-squared tests for *p*-values and percentages (95% CI). The independent associations among niacin intake, CVD, and all-cause mortality were assessed using multivariate Cox regression tests. To explore the sustainability of our outcomes, we conducted sensitivity analyses using extended Cox regression models with different covariate adjustments. Log-rank tests and Kaplan–Meier survival curves were generated using R software (version 4.3.1, R Foundation for Statistical Computing, Vienna, Austria) to compare survival probabilities between groups [25]. Three models were used to address potential confounding factors: Model 1 had no adjustments; Model 2 controlled for age, sex, and race; and Model 3 included all variables from Model 2, along with poverty income ratio, education, marital status, diabetes, smoking, BMI, waist circumference, alcohol consumption, and hypertension. Notably, sex adjustment was applied only to the total population analysis. Finally, restricted cubic splines were used to assess the potential nonlinear relationships. R software (version 4.3.1) and Empower 6.0 (http://www.empowerstats.com, X&Y Solutions, Inc., Boston, MA, USA) were used for each analysis, with significance set at *p* < 0.05.

## 3. Results

### 3.1 Baseline Features

The NHANES 2003–2018 sample initially included 80,312 participants. After excluding participants with missing niacin data, the sample size was reduced to 60,813. Further exclusions of patients with missing mortality and CVD morbidity records reduced the eligible sample size to 34,828 patients for analysis. Among them, 16,557 were males, and 18,271 were females. Participants were stratified into four categories based on the amount of niacin consumed each day: Q1 (<14.646 mg, n = 8666), Q2 (14.646–21.302 mg, n = 8824), Q3 (21.302–30.401 mg, n = 8704), and Q4 (>30.401 mg, n = 8824). The baseline characteristics of the patients are summarised in Table 1. Interestingly, the participants in the group with the highest niacin intake tended to be younger. Additionally, a noteworthy observation was the higher representation of females in the higher niacin intake quartiles compared to males, with the reverse trend observed in the lower quartile. The characteristics of those in the highest niacin intake quartile included being married, non-smoker, non-Hispanic white, and less likely to have ‘less than high school education’. Conversely, individuals in the lowest intake group were more likely to have a history of diabetes, CVD, and hypertension, although their BMI and alcohol intake were not substantially different from those in the other groups

**Table 1.** **Baseline characteristics of the study population stratifed by dietary niacin intake**.

Variable		Total (n = 34,828)	Q1 (n = 8666)	Q2 (n = 8824)	Q3 (n = 8704)	Q4 (n = 8634)	*p*-value
Age (years)		47.3 (46.9, 47.8)	49.0 (48.4, 49.7)	48.9 (48.3, 49.6)	47.5 (46.9, 48.2)	44.4 (43.8, 45.0)	<0.0001
Poverty income ratio		3.0 (2.9, 3.1)	2.7 (2.6, 2.8)	3.0 (3.0, 3.1)	3.1 (3.0, 3.1)	3.2 (3.1, 3.3)	<0.0001
Body mass index (kg/m^2^)		29.0 (28.8, 29.1)	29.1 (28.9, 29.4)	28.9 (28.6, 29.1)	29.1 (28.8, 29.3)	28.9 (28.6, 29.1)	0.1738
Waist circumference (cm)		99.0 (98.6, 99.4)	98.4 (97.8, 99.0)	98.4 (97.9, 99.0)	99.4 (98.8, 100.0)	99.6 (99.0, 100.2)	0.005
Avg alcoholic drinks/day (12 Mos)		3.3 (3.0, 3.6)	3.8 (2.9, 4.8)	3.1 (2.6, 3.6)	3.1 (2.7, 3.5)	3.3 (3.0, 3.5)	0.5477
Gender							<0.0001
	Female	52.3 (51.6, 53.0)	71.2 (69.8, 72.6)	62.7 (61.3, 64.1)	49.0 (47.5, 50.5)	31.4 (30.2, 32.7)	
	Male	47.7 (47.0, 48.4)	28.8 (27.4, 30.2)	37.3 (35.9, 38.7)	51.0 (49.5, 52.5)	68.6 (67.3, 69.8)	
Race							<0.0001
	Mexican American	8.5 (7.3, 9.8)	9.6 (8.1, 11.4)	8.4 (7.2, 9.9)	7.8 (6.6, 9.2)	8.2 (7.0, 9.5)	
	Other Hispanic	5.1 (4.3, 5.9)	5.9 (4.9, 7.1)	4.9 (4.1, 5.8)	4.7 (4.0, 5.5)	4.9 (4.1, 5.9)	
	Non-Hispanic White	68.1 (65.6, 70.4)	63.4 (60.4, 66.4)	68.0 (65.2, 70.7)	69.9 (67.2, 72.4)	70.0 (67.6, 72.4)	
	Non-Hispanic Black	11.3 (10.1, 12.7)	14.4 (12.8, 16.2)	11.1 (9.8, 12.6)	10.6 (9.3, 12.1)	9.8 (8.6, 11.2)	
	Other race	7.1 (6.4, 7.8)	6.6 (5.7, 7.6)	7.6 (6.6, 8.7)	7.0 (6.1, 8.1)	7.1 (6.3, 8.0)	
Education (years)							<0.0001
	Less than 9th grade	5.0 (4.5, 5.5)	8.1 (7.2, 9.1)	5.5 (4.8, 6.3)	4.1 (3.6, 4.7)	3.0 (2.6, 3.5)	
	9–11th grade	10.3 (9.6, 11.1)	13.2 (12.1, 14.5)	10.1 (9.0, 11.2)	8.9 (7.9, 9.9)	9.5 (8.7, 10.4)	
	High school grad/GED or equivalent	23.6 (22.6, 24.5)	24.8 (23.4, 26.4)	23.2 (21.7, 24.7)	24.4 (22.8, 26.0)	22.2 (20.5, 23.9)	
	Some college or AA degree	31.7 (30.7, 32.6)	30.1 (28.5, 31.7)	31.4 (29.7, 33.2)	31.6 (30.0, 33.3)	33.1 (31.8, 34.6)	
	College graduate or above	29.4 (27.8, 31.1)	23.7 (21.7, 25.9)	29.8 (27.5, 32.2)	31.0 (28.8, 33.2)	32.2 (30.1, 34.3)	
	Missing	0.0 (0.0, 0.1)	0.0 (0.0, 0.1)	0.0 (0.0, 0.1)	0.1 (0.0, 0.2)	0.0 (0.0, 0.1)	
Marriage							<0.0001
	Married	55.5 (54.1, 56.8)	50.3 (48.5, 52.1)	57.9 (55.8, 59.9)	57.4 (55.4, 59.4)	55.6 (53.7, 57.5)	
	Widowed	5.8 (5.4, 6.2)	8.8 (8.1, 9.6)	6.7 (6.1, 7.5)	5.2 (4.6, 5.9)	3.1 (2.7, 3.7)	
	Divorced	10.2 (9.7, 10.7)	11.6 (10.6, 12.7)	9.6 (8.7, 10.6)	9.9 (9.0, 10.9)	9.8 (8.8, 10.9)	
	Separated	2.3 (2.1, 2.5)	2.9 (2.4, 3.4)	2.3 (1.9, 2.7)	2.0 (1.6, 2.4)	2.1 (1.7, 2.6)	
	Never married	18.4 (17.3, 19.6)	18.5 (17.0, 20.1)	16.2 (14.7, 17.9)	17.7 (16.1, 19.4)	20.9 (19.3, 22.6)	
	Living with partner	7.8 (7.3, 8.4)	7.8 (6.9, 8.8)	7.3 (6.4, 8.3)	7.7 (6.8, 8.7)	8.3 (7.4, 9.3)	
	Missing	0.0 (0.0, 0.1)	0.0 (0.0, 0.1)	0.0 (0.0, 0.0)	0.0 (0.0, 0.1)	0.1 (0.0, 0.2)	
Smoking							<0.0001
	Not at all	24.9 (24.1, 25.8)	22.9 (21.5, 24.4)	24.1 (22.5, 25.8)	25.5 (24.0, 27.1)	26.6 (25.1, 28.2)	
	Some days	3.7 (3.4, 4.0)	3.5 (3.0, 4.1)	2.9 (2.4, 3.5)	3.5 (3.0, 4.1)	4.7 (4.0, 5.4)	
	Every day	16.4 (15.5, 17.3)	19.8 (18.4, 21.4)	15.3 (14.0, 16.7)	15.2 (14.1, 16.5)	15.7 (14.5, 17.0)	
	Missing	55.0 (53.9, 56.1)	53.7 (51.8, 55.6)	57.7 (55.9, 59.4)	55.8 (54.1, 57.4)	53.0 (51.2, 54.8)	
Hypertension							0.0286
	No	61.9 (60.9, 62.8)	60.1 (58.3, 61.8)	61.4 (59.8, 63.0)	62.6 (60.9, 64.2)	63.0 (61.4, 64.6)	
	Yes	35.3 (34.4, 36.2)	36.8 (35.1, 38.4)	35.3 (33.8, 36.7)	34.9 (33.3, 36.6)	34.5 (33.0, 36.0)	
	Missing	2.9 (2.5, 3.3)	3.2 (2.7, 3.8)	3.3 (2.6, 4.3)	2.5 (2.1, 3.0)	2.6 (2.1, 3.0)	
Cardiovascular disease (CVD)							<0.0001
	No	91.2 (90.6, 91.7)	89.1 (88.0, 90.1)	90.6 (89.7, 91.3)	90.9 (89.9, 91.7)	93.6 (92.8, 94.3)	
	Yes	8.8 (8.3, 9.4)	10.9 (9.9, 12.0)	9.4 (8.7, 10.3)	9.1 (8.3, 10.1)	6.4 (5.7, 7.2)	
Diabetes							<0.0001
	No	88.8 (88.3, 89.4)	87.2 (86.2, 88.2)	87.8 (86.8, 88.7)	89.3 (88.3, 90.2)	90.6 (89.8, 91.4)	
	Yes	9.3 (8.8, 9.8)	10.6 (9.7, 11.6)	10.2 (9.4, 11.2)	9.1 (8.3, 10.0)	7.5 (6.9, 8.2)	
	Borderline	1.9 (1.7, 2.1)	2.1 (1.7, 2.5)	2.0 (1.5, 2.5)	1.6 (1.3, 2.0)	1.9 (1.5, 2.3)	
	Missing	0.0 (0.0, 0.0)	0.0 (0.0, 0.1)	0.0 (0.0, 0.0)	0.0 (0.0, 0.0)	0.0 (0.0, 0.1)	

Data in the table: For continuous variables: survey-weighted mean (95% CI); For categorical variables: survey-weighted percentage (95% CI). Mos, months; GED, general educational development; AA, Associate of Arts; CI, confidence interval; Avg, average.

### 3.2 Association between Niacin and the Prevalence of CVD

**Fig. 2.**
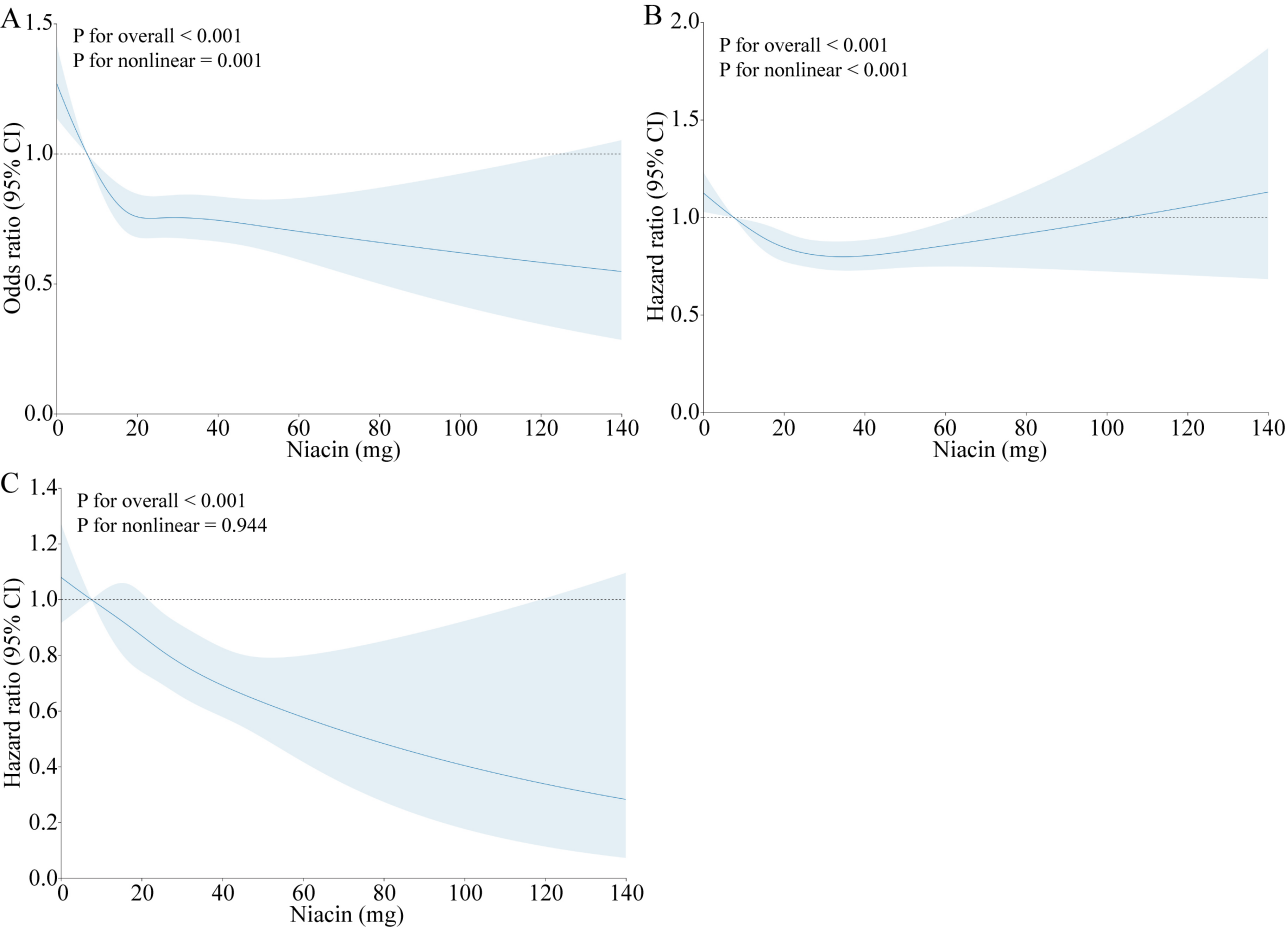
**Dose–response relationship between the prevalence of CVD (A), all-cause mortality (B), CVD mortality (C), and dietary niacin intake**. Adjusted for age, sex, race, education, poverty income ratio, marriage, diabetes, smoking, BMI, waist circumference, alcohol consumption, and hypertension. CVD, cardiovascular disease; BMI, body mass index; CI, confidence interval.

After adjusting for various factors, including race, socioeconomic status (poverty-income ratio), education, marital status, diabetes, sex, smoking habits, BMI, age, alcohol consumption, waist circumference, and hypertension, a significant non-linear relationship emerged between niacin intake levels and the prevalence of CVD in the entire population (*p* < 0.001 for nonlinearity; Fig. 2A). As shown in Table 2, compared to those of the reference group (Q1), the odds ratios (ORs) for CVD prevalence in the total population were 0.91 (95% CI: 0.80–1.05), 0.97 (95% CI: 0.82–1.13), and 0.80 (95% CI: 0.67–0.96) in Q2, Q3, and Q4 groups. The highest niacin intake level (Q4) was notably associated with a reduced CVD prevalence. Subsequent investigation showed comparable trends between sexes. Males in Q2, Q3, and Q4 had ORs of 1.00 (95% CI: 0.81–1.25), 1.01 (95% CI: 0.79–1.28), and 0.84 (95% CI: 0.67–1.05) for CVD prevalence, respectively, compared to those in Q1. Similarly, females in Q2, Q3, and Q4 had ORs of 0.85 (95% CI: 0.73–1.01), 0.93 (95% CI: 0.76–1.14), and 0.81 (95% CI: 0.61–1.08), respectively, compared to the reference group (Q1).

**Table 2.** **The relationship between dietary niacin intake and the prevalence of cardiovascular disease (CVD)**.

Participates	Niacin classification	Model 1		Model 2		Model 3	
		OR (95% CI)	*p*-value	OR (95% CI)	*p*-value	OR (95% CI)	*p*-value
Total	Q1	1 [Reference]		1 [Reference]		1 [Reference]	
	Q2	0.85 (0.75, 0.97)	0.013	0.84 (0.73, 0.97)	0.015	0.91 (0.80, 1.05)	0.2
	Q3	0.82 (0.72, 0.94)	0.005	0.88 (0.76, 1.03)	0.1	0.97 (0.82, 1.13)	0.7
	Q4	0.56 (0.48, 0.65)	<0.001	0.71 (0.59, 0.84)	<0.001	0.80 (0.67, 0.96)	0.016
Male	Q1	1 [Reference]		1 [Reference]		1 [Reference]	
	Q2	0.89 (0.73, 1.08)	0.2	0.93 (0.74, 1.16)	0.5	1 (0.81, 1.25)	>0.9
	Q3	0.74 (0.61, 0.91)	0.003	0.92 (0.73, 1.16)	0.5	1.01 (0.79, 1.28)	>0.9
	Q4	0.46 (0.38, 0.56)	<0.001	0.73 (0.58, 0.92)	0.008	0.84 (0.67, 1.05)	0.13
Female	Q1	1 [Reference]		1 [Reference]		1 [Reference]	
	Q2	0.77 (0.65, 0.91)	0.002	0.78 (0.66, 0.93)	0.004	0.85 (0.73, 1.01)	0.061
	Q3	0.76 (0.63, 0.91)	0.004	0.86 (0.70, 1.06)	0.15	0.93 (0.76, 1.14)	0.5
	Q4	0.52 (0.40, 0.67)	<0.001	0.73 (0.55, 0.97)	0.029	0.81 (0.61, 1.08)	0.14

Model 1 was adjusted for none. Model 2 was adjusted for age, sex, and race. Model 3 was adjusted for age, sex, race, education, poverty-to-income ratio, marriage, diabetes, smoking, BMI, waist circumference, alcohol consumption, and hypertension. OR, odds ratio; CI, confidence interval; BMI, body mass index. For male and female, sex was not adjusted.

### 3.3 Relationship between Niacin and Death from All Causes

**Fig. 3.**
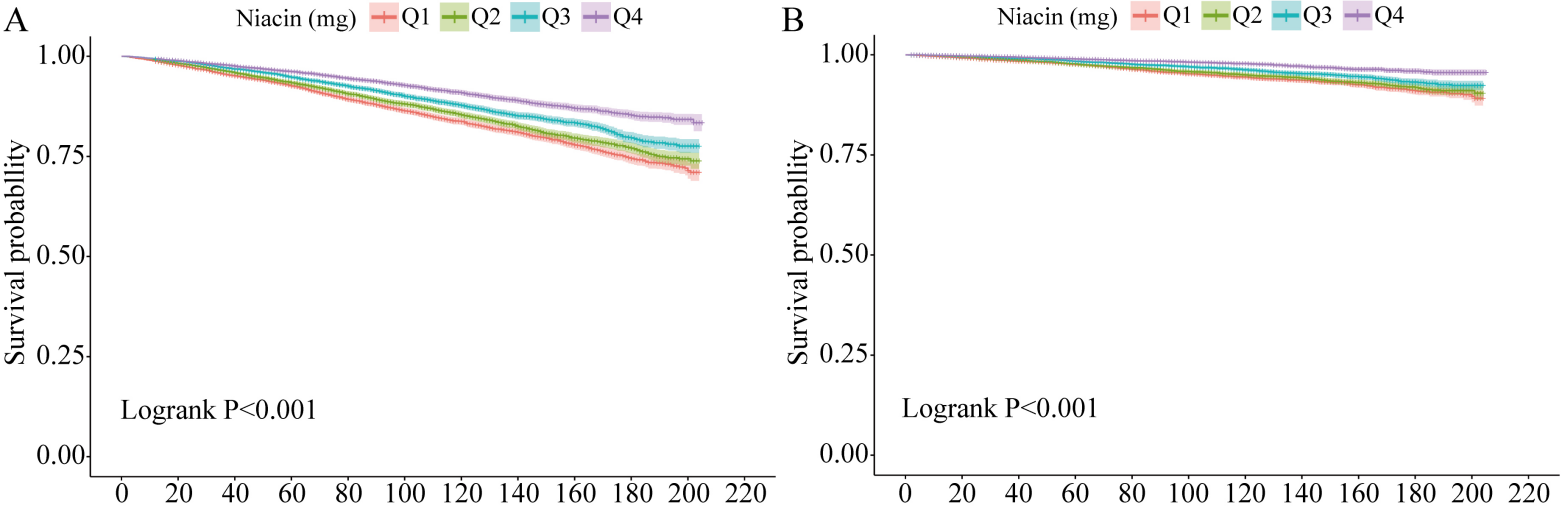
**Kaplan–Meier survival curves for mortality outcomes**. (A) for all-cause mortality, (B) for CVD mortality. CVD, cardiovascular disease.

Significant differences in cardiovascular mortality were observed among the Q1, Q2, Q3, and Q4 categories (Log-rank *p* < 0.001; Fig. 3A). Q1 had the lowest survival rate (Fig. 3A). A nonlinear relationship was found between CVD mortality and niacin intake levels (*p* < 0.001 for nonlinearity; Fig. 2B). After accounting for covariates in Table 3, the hazard ratios (HRs) for all-cause mortality in the whole population were 0.91 (95% CI: 0.82–1.02) in Q2, 0.96 (95% CI: 0.83–1.10) in Q3, and 0.82 (95% CI: 0.71–0.96) in Q4 compared to Q1. Males in Q2, Q3, and Q4 had HRs of 0.92 (95% CI: 0.77–1.10), 0.99 (95% CI: 0.82–1.21), and 0.86 (95% CI: 0.69–1.05), respectively, compared to males in Q1. Likewise, females in Q2, Q3, and Q4 had HRs of 0.92 (95% CI: 0.78–1.07), 0.93 (95% CI: 0.78–1.11), and 0.78 (95% CI: 0.62–0.97) compared to Q1. Notably, a lower risk of all-cause death in females was linked to greater niacin intake (≥30.401 mg/d).

**Table 3.** **Relationship between dietary niacin intake and all-cause mortality**.

Participates	Niacin classification	Model 1		Model 2		Model 3	
		HR (95% CI)	*p*-value	HR (95% CI)	*p*-value	HR (95% CI)	*p*-value
Total	Q1	1 [Reference]		1 [Reference]		1 [Reference]	
	Q2	0.85 (0.76, 0.96)	0.01	0.83 (0.74, 0.93)	0.002	0.91 (0.82, 1.02)	0.12
	Q3	0.8 (0.70, 0.92)	0.002	0.85 (0.74, 0.98)	0.023	0.96 (0.83, 1.10)	0.5
	Q4	0.52 (0.45, 0.60)	<0.001	0.71 (0.61, 0.83)	<0.001	0.82 (0.71, 0.96)	0.012
Male	Q1	1 [Reference]		1 [Reference]		1 [Reference]	
	Q2	0.78 (0.65, 0.94)	0.008	0.83 (0.70, 0.99)	0.039	0.92 (0.77, 1.10)	0.4
	Q3	0.68 (0.57, 0.82)	<0.001	0.86 (0.71, 1.04)	0.12	0.99 (0.82, 1.21)	>0.9
	Q4	0.42 (0.34, 0.51)	<0.001	0.7 (0.57, 0.86)	<0.001	0.86 (0.69, 1.05)	0.14
Female	Q1	1 [Reference]		1 [Reference]		1 [Reference]	
	Q2	0.85 (0.71, 1.01)	0.071	0.84 (0.72, 0.98)	0.025	0.92 (0.78, 1.07)	0.3
	Q3	0.77 (0.64, 0.94)	0.009	0.84 (0.70, 1.00)	0.054	0.93 (0.78, 1.11)	0.4
	Q4	0.47 (0.37, 0.59)	<0.001	0.72 (0.57, 0.89)	0.003	0.78 (0.62, 0.97)	0.028

Model 1 was adjusted for none. Model 2 was adjusted for age, sex, and race. Model 3 was adjusted for age, sex, race, education, poverty-to-income ratio, marriage, diabetes, smoking, BMI, waist circumference, alcohol consumption, and hypertension. HR, hazard ratio; CI, confidence interval; BMI, body mass index. For male and female, sex was not adjusted.

### 3.4 Association of Niacin with CVD Mortality

Fig. 3B shows the Kaplan–Meier survival curve, revealing significant differences in survival time among participants with varying niacin intake levels (log-rank test, *p* < 0.001). Fully adjusted restricted cubic spline regression analysis (Fig. 2C) illustrated an inverse U-shaped association between niacin intake and cardiovascular mortality (non-linear, *p* < 0.001). After adjusting for multiple variables (Table 4), the HRs for CVD mortality in the entire population were as follows: 0.99 (95% CI: 0.79–1.23) in Q2, 0.97 (95% CI: 0.78–1.20) in Q3, and 0.87 (95% CI: 0.67–1.13) in Q4 compared to those in Q1. Male individuals in quartiles Q2, Q3, and Q4 had HRs of 1.09 (95% CI: 0.78–1.52), 1.16 (95% CI: 0.84–1.61), and 0.98 (95% CI: 0.69–1.37) respectively, compared to those of the reference group. Women had HRs of 0.92 (95% CI: 0.76–1.11) in the second quartile, 0.75 (95% CI: 0.59–0.94) in the third quartile, and 0.70 (95% CI: 0.51–0.97) in the fourth quartile. A higher niacin intake of 21.302 mg/day was associated with a decreased risk of all-cause mortality in females.

**Table 4.** **Relationship between dietary niacin intake and CVD mortality**.

Participates	Niacin classification	Model 1		Model 2		Model 3	
		HR (95% CI)	*p*-value	HR (95% CI)	*p*-value	HR (95% CI)	*p*-value
Total	Q1	1 [Reference]		1 [Reference]		1 [Reference]	
	Q2	0.92 (0.74, 1.15)	0.5	0.91 (0.74, 1.13)	0.4	0.99 (0.79, 1.23)	>0.9
	Q3	0.78 (0.63, 0.96)	0.022	0.87 (0.70, 1.07)	0.2	0.97 (0.78, 1.20)	0.7
	Q4	0.48 (0.37, 0.63)	<0.001	0.74 (0.57, 0.97)	0.029	0.87 (0.67, 1.13)	0.3
Male	Q1	1 [Reference]		1 [Reference]		1 [Reference]	
	Q2	0.91 (0.67, 1.24)	0.6	0.97 (0.71, 1.33)	0.9	1.09 (0.78, 1.52)	0.6
	Q3	0.76 (0.56, 1.02)	0.063	0.99 (0.72, 1.36)	>0.9	1.16 (0.84, 1.61)	0.4
	Q4	0.42 (0.31, 0.58)	<0.001	0.78 (0.56, 1.08)	0.14	0.98 (0.69, 1.37)	0.9
Female	Q1	1 [Reference]		1 [Reference]		1 [Reference]	
	Q2	0.81 (0.68, 0.98)	0.033	0.87 (0.72, 1.05)	0.2	0.92 (0.76, 1.11)	0.4
	Q3	0.56 (0.45, 0.70)	<0.001	0.7 (0.56, 0.88)	0.002	0.75 (0.59, 0.94)	0.012
	Q4	0.35 (0.25, 0.48)	<0.001	0.67 (0.48, 0.92)	0.013	0.70 (0.51, 0.97)	0.032

Model 1 was adjusted for none. Model 2 was adjusted for age, sex, and race. Model 3 was adjusted for age, sex, race, education, poverty-to-income ratio, marriage, diabetes, smoking, BMI, waist circumference, alcohol consumption, and hypertension. HR, hazard ratio; CI, confidence interval; CVD, cardiovascular disease; BMI, body mass index. For male and female, sex was not adjusted.

## 4. Discussion

Our study revealed favorable associations between niacin and cardiovascular events using data from the NHANES 2003–2018. We identified a negative correlation between the population’s prevalence of CVD and niacin levels, which persisted after adjusting for covariates, suggesting niacin’s protective role against CVD development. The cardiovascular protective effect may stem from niacin’s ability to decrease LDL-C and triglycerides and improve lipoprotein function [26]. A meta-analysis published in 2006, encompassing 23 studies, indicated that for every 1% decrease in LDL-C, the incidence of CVD events decreased by nearly 1%, and for every 1% increase in HDL-C, the incidence of CVD events decreased by at least 1%, irrespective of LDL-C reduction [27]. Dose–response analysis revealed a statistically significant and nonlinear trend, with a sharp decline in the ORs at lower doses and a plateau at higher doses. While sex-based differences in CVD incidence were not substantial, a discernible, though statistically non-significant, trend towards reduced CVD prevalence with niacin therapy was observed. While research on the link between niacin and CVD is ongoing, interest in using dietary niacin as an intervention is growing. The Landmark Coronary Drug Project (1966–1975) explored the long-term reliability and effectiveness of niacin and other lipid-altering drugs [28, 29]. Although it showed modest benefits in reducing nonfatal heart attacks, the lack of impact on overall mortality led to the trial being considered neutral and overlooked [28]. In a placebo-controlled study involving 1119 male patients administered 2000 mg of niacin daily and 2789 taking a placebo, the niacin group showed a 26% lower rate of nonfatal heart attacks and a 24% decrease in strokes (*p* < 0.05) [11]. A subsequent meta-analysis of seven trials confirmed momentous drops in stroke, nonfatal heart attacks, coronary revascularization, and transient ischaemic attacks with niacin compared to placebo [30]. Interestingly, it also hinted at a potential but non-significant decrease in cardiac mortality (*p* = 0.13). It should be noted that the niacin doses in these trials varied [30]. Our investigation examined participants’ baseline and nutritional characteristics based on niacin intake. Despite controlling for several variables, there was no correlation with a lower risk of all-cause or CVD mortality in men. Similarly, a meta-regression analysis of 11 niacin trials [31] linked serum HDL-C levels with a lower probability of CVDs. This may be owing to the independent anti-atherogenic effects of niacin through its antioxidant and anti-inflammatory properties [32].

In a 2011 randomised controlled trial (RCT), 3414 patients diagnosed with coronary heart disease who underwent simvastatin treatment were randomly allocated to either the niacin or placebo group [33]. The participants received simvastatin at doses ranging from 40 to 80 mg, with or without ezetimibe at 10 mg/d, to attain LDL-C levels between 40 and 80 mg/dL. After a 3-year follow-up, no significant advantage was observed in the primary endpoint of composite CVD (95% CI: 0.87–1.21, HR: 1.02; *p* = 0.79). However, the niacin cohort exhibited a notable elevation in median HDL-C levels compared to those of the placebo group and reductions in triglyceride and LDL-C levels compared to those at baseline. Furthermore, a random-effects meta-analysis of 23 RCTs indicated that niacin did not significantly affect mortality but was associated with adverse outcomes. These included the frequency of nonfatal or fatal infarctions of the heart, cardiovascular or non-cardiovascular death, and the frequency of nonfatal or fatal strokes [26]. A recent study reported that niacin did not negatively affect cardiovascular outcomes during secondary prevention [34]. Moreover, in individuals not taking statin medication, niacin monotherapy was associated with a lower risk of cardiovascular events (relative risk: 0.51, 95% CI: 0.37–0.72; proportional risk: 0.74, 95% CI: 0.58–0.96; stroke: relative risk: 0.74, 95% CI: 0.59–0.94; acute coronary syndrome: and revascularization) [34]. This study did not demonstrate a link between niacin intake and a decreased risk of cardiovascular morbidity or death. However, the sex-stratified analysis revealed a positive association between niacin intake and both CVD and all-cause mortality. Interestingly, females appeared to benefit from higher niacin intake levels, as evidenced by a lack of marked variability in CVD and all-cause mortality compared to males. Further studies are necessary to understand the root cause of this disparity in the impact of niacin consumption based on sex.

Clinical trials have identified various adverse effects associated with niacin, ranging from common symptoms, such as skin flushing and itching, to more severe conditions, such as heart failure, musculoskeletal and gastrointestinal issues, diabetic complications, and new-onset diabetes [30]. Despite these side effects, niacin remains the most potent agent currently available for enhancing HDL levels, exhibiting the ability to increase HDL levels by as much as 30–35%, even in individuals with extremely low HDL levels [35]. Surjana *et al*. [36] demonstrated the role of niacin in inhibiting carcinogenesis and DNA damage in various cancers, including oral, colon, breast, and lung cancers. Recent clinical research has revealed a correlation between niacin consumption and a lower risk of developing squamous cell carcinoma [37]. These findings hold significance for clinicians for several reasons. Firstly, statin-treated patients often face a residual risk of CVD. Secondly, evidence suggests that improving prognosis beyond lowering LDL-C levels may be beneficial. Furthermore, niacin could be an appealing alternative for an estimated 10% of patients who cannot tolerate or have contraindications to statin therapy [38]. Thirdly, research suggests females may benefit more than males from higher niacin intake levels in reducing CVD mortality. The present study had several limitations. Firstly, potential errors and inaccuracies in diet assessments could introduce uncertainty. Secondly, the presence of residual confounding factors is inherent in observational studies. Although adjustments have been made to various covariates to minimise this issue, complete elimination remains challenging. Thirdly, the self-reported 24-h recalls used to collect dietary data might be susceptible to recall bias. While this method is widely employed by trained interviewers [39, 40], it is essential to acknowledge this limitation. Finally, the scarcity of comprehensive CVD data, particularly regarding mortality data, owing to the relatively small number of deaths is recognised. Large-scale investigations such as clinical studies can provide substantial evidence of the relationship under investigation.

## 5. Conclusions

Increased dietary niacin intake is associated with CVD incidence but does not show a substantial correlation with all-cause mortality in the overall population. However, it is noteworthy that an intake of 30.401 mg/d of niacin lowers the risk of all-cause death in females. In addition, a higher niacin intake of 21.302 mg/d or more appears to have a protective effect against CVD mortality, specifically in females, but with no such observed impact in males. Overall, increasing niacin supplementation may help reduce cardiovascular risk in high-risk female patients. However, further prospective studies are required to clarify whether increased niacin intake reduces the risk of CVD mortality in females.

## Availability of Data and Materials

All data generated or analyzed during this study are included in this published article.

## Supplementary Material

Supplementary material associated with this article can be found, in the online version, at https://doi.org/10.31083/j.rcm2511410.
